# Long-term outcomes of macrovascular diseases and metabolic indicators of bariatric surgery for severe obesity type 2 diabetes patients with a meta-analysis

**DOI:** 10.1371/journal.pone.0224828

**Published:** 2019-12-03

**Authors:** Guoli Yan, Jinjin Wang, Jianfeng Zhang, Kaiping Gao, Qianqian Zhao, Xueqin Xu

**Affiliations:** 1 Public Health and Preventive Medicine Teaching and Research Center, Henan University of Chinese Medicine, Zhengzhou, People’s Republic of China; 2 Henan Armed Police Corps Hospital, Zhengzhou, People’s Republic of China; 3 Department of Preventive Medicine, Shenzhen University Health Science Center, Shenzhen, Guangdong, China; Hopital Europeen Georges Pompidou, FRANCE

## Abstract

There is currently no detailed evidence for the long-term effects of bariatric surgery on severely obese with type 2 diabetes, such as the risk of myocardial infarction and stroke. In order to provide evidence on the risks of macrovascular diseases and metabolic indicators of bariatric surgery follow-up for more than five years, we searched in the Cochrane library, Pubmed, and EMBASE databases from the earliest studies to January 31, 2019. Randomized clinical trials or cohort studies compared bariatric surgery and conventional medical therapy for long-term incidence of macrovascular events and metabolic outcomes in severely obese patients with T2DM. Fixed-effects and random-effects meta-analyses were performed to pool the relative risks (RRs), hazard ratios (HRs) and weighted mean difference (WMD). Publication bias and heterogeneity were examined. Four RCTs and six cohort studies were finally involved in this review. Patients in the bariatric surgery group as compared to the conventional treatment group had lower incidence of macrovascular complications (RR = 0.43, 95%CI = 0.27~0.70), cardiovascular events (CVEs) (HR = 0.52, 95%CI = 0.39~0.71), and myocardial infarction (MI) (RR = 0.40, 95%CI = 0.26~0.61). At the same time, the results demonstrate that bariatric surgery is associated with better weight and better glycemic control over the long-term than non-surgical therapies, and reveal that different surgical methods have different effects on various metabolic indicators. Bariatric surgery significantly decreases macrovascular complications over the long term and is associated with greater weight loss and better intermediate glucose outcomes among T2DM patients with severe obesity as compared to patients receiving only conservative medical measures.

## Introduction

Type 2 diabetes mellitus (T2DM) is a chronic, non-communicable disease caused by the inability of the pancreas to produce enough insulin or the body’s inability to use insulin effectively, which can cause micro- and macrovascular complications [1]. The increased social and economic burdens associated with T2DM make prevention of obesity and T2DM urgently needed.

Recently published randomized controlled trials (RCTs) recommend bariatric surgery as the best treatment for obesity and T2DM [2]. These meta-analyses measured effectiveness outcome indicators including weight loss and blood glucose levelswithin 2 years [3–5]. Two earlier meta-analyses had shown that bariatric surgery had significantly higher diabetes remission rates over 5 years [6, 7]. A recent meta-analysis published in 2017 suggested better remission and lower risks of microvascular and macrovascular complications in the bariatric surgery as compared to non-surgical treatment group based on studies reported prior to 2016 [8]. However, meta-analyses published to date have not systematized the evidence on the long-term which means followed up for more than five years effects of the various surgical types and specific outcomes such as myocardial infarction (MI) and stroke on T2DM with severe obesity.

Therefore, the objective of this study was to evaluate the macrovascular diseases, including coronary artery disease and cerebrovascular disease, and separately MI and stroke, and the physiological and biochemical metabolic indicators following bariatric surgery with comparison to non-surgical treatments in severely obese patients with T2DM.

## Methods

This review addressed 2 key questions:

**Key question 1.** Does bariatric surgery decrease macrovascular events and mortality in severely obese patients with type 2 diabetes after at least five years of follow-up?
**Key question 2.** What is the impact of bariatric surgery on metabolic outcomes in severely obese patients with type 2 diabetes?

### Data sources and searches

We conducted our systematic literature search in the Cochrane library, Pubmed, and EMBASE databases from the earliest studies to January 31, 2019, using the combination of Medical terms (MeSH) and keywords for T2DM and bariatric surgery as the search strategy: for Cochrane library using MeSH of ‘*Bariatric Surgery*’, ‘*Diabetes Mellitus*, *Type 2’* and their related Exact Term Match words as keywords; for Pubmed databases, using the MeSH of ‘*Bariatric Surgery’*, ‘*Diabetes Mellitus*, *Type 2’* and their related entry terms as keywords; and for EMBASE databases, using Emtree of ‘*bariatric surgery*’,. ‘*non insulin dependent diabetes mellitus*’ and their related Synonyms as keywords. In addition, we used the keywords ‘macrovascular events or coronary artery disease or cerebrovascular diseases or macrovascular complications or mortality or follow-up or longitudinal studies or outcome assessment or randomized controlled trial’. The search range was within the full text, and the language was limited to English. The search result included in 5275 published related literature (including review articles, abstracts from conferences, original papers and so on) after removal of duplicates, which were subjected to further screening via the following inclusion criteria.

### Inclusion criteria

Studies were selected based on the following inclusion criteria: (1) RCT or cohort studies; (2) comparison of bariatric surgery including Roux-en-Y gastric bypass (RYGB), adjustable gastric banding (AGB), sleeve gastrectomy (SG), vertical banded gastroplasty (VBG), biliopancreatic diversion (BPD) to conventional medical therapy; (3) reported at least one of the main outcomes of interest (macrovascular events, mortality, or metabolic outcomes such as glucose, HbA_1c_%, HOMA-IR, blood pressure, lipids, weight and BMI); (4) patient follow-up beyond 5 years; (5) studies enrolling adults baseline BMI ≥ 35. If there were separate publications from the same study, we selected the one with the most updated data.

### Exclusion criteria

**Fig 1:**
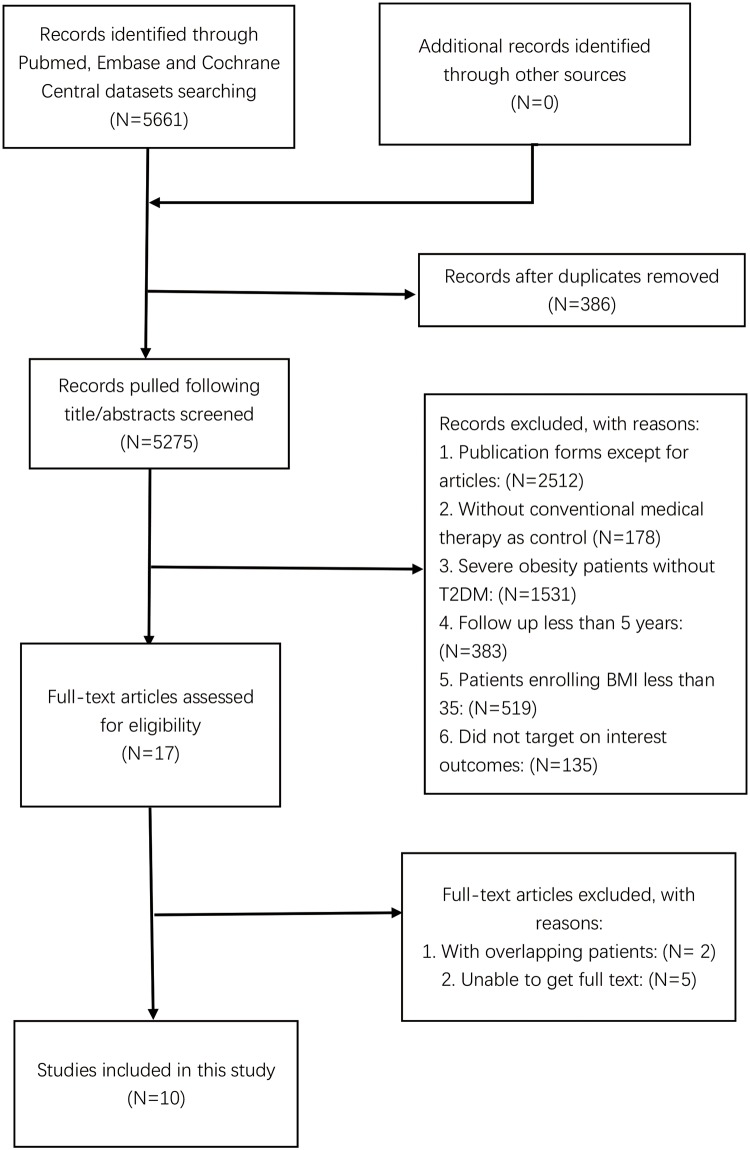
Literature search and screening process.

Exclusion criteria were as follows: (1) trials without conventional medical therapy as control (N = 178); (2) severely obese patients without T2DM (N = 1531); (3) follow up less than 5 years (N = 383); (4) patients with BMI less than 35 (N = 519); (5) did not target our interest outcomes (N = 135); (6) publication forms other than peer-reviewed articles (N = 2512). We excluded 5 studies because of a lack of full text and 2 studies with overlapping patients. A total of 10 eligible publications were included in the meta-analysis [9–18] (Fig 1).

### Risk-of-bias assessment

We used Cochrane risk-of-bias criteria to examine the methodological quality for the included RCTs [19], and each quality item was graded as unclear risk, high risk, and low risk. The seven quality items used to examine bias in each trial included randomization sequence generation, allocation concealment, blinding of participants and personnel, blinding of outcome assessment, incomplete outcome data, selective reporting, and other bias (defined as early termination of research for data reason or reason for formal termination, or significant imbalance in baseline). S1 Fig lists the results of risk-of–bias assessment by Review Manager 5.2 (S1 Fig). S1 Table lists the results of OTTAWA quality assessment scale of cohort studies (S1 Table).

### Data extraction

Data were independently extracted by two reviewers using a standardized data extraction form for each study. Discrepancies were adjudicated by the third reviewer until a consensus was achieved on each item. The following data were extracted from the eligible articles: first author’s name, publication date, study country, study design, surgery type, main outcomes, follow-up years, the sample size, BMI at baseline, age at baseline, the amount of macrovascular complications or all-cause mortality, or adjusted HRs and 95% CI. Two additional authors checked on information accuracy from the original manuscripts.

### Data synthesis and statistical analysis

All meta-analyses were performed with the R 3.5.2 and STATA 15.0 software package. All tests were 2-tailed, and *P*≤ 0.05 was considered statistically significant. For KQ1, the strength of the effects between bariatric surgery and severe obesity with T2DM was measured by determining relative risk (RR) with 95% CIs (patient numbers provided in the original literature) or HRs with 95%CIs (adjusted HRs provided in the original literature). For KQ2, the metabolic outcomes, random effects meta-analyses were conducted to calculate the pooled weighted mean difference (WMD) and 95% CIs for continuous data.

We used the heterogeneity index (*I*^2^, 0–100) to assess heterogeneity among the studies [20], and accepted a high degree of heterogeneity for study pairs with an *I*^2^>50% in accordance with the Cochrane reviewer’s handbook 5.1.0 [19]. Baujat diagrams can be used to analyze studies that over-contribute to heterogeneity and overall results [21]. The horizontal axis of the graph represents the heterogeneity of the study, while the vertical axis represents the impact of the study on the overall results. When heterogeneity was not an issue, the fixed effects model was applied with the Mantel-Haenszel method [22]. Otherwise, a random effects model was applied using the DerSimonian and Laird method to calculate the combined RR or HR [23]. For continuous data, the method of Inverse Variance was used for fixed effects model, and I-V heterogeneity was used for random effects model.

Publication bias was investigated with funnel plots [24]. Furthermore, Egger’s regression approach was adopted [25]. The sensitivity analysis was conducted in which each study was excluded one by one, to evaluate the influence of that particular study on the overall estimates.

## Results

### Studies characteristics

Four RCTs [10, 13, 15, 18] and six cohort studies (three retrospective cohorts [11, 14, 17], and three prospective cohorts [9, 12, 16]) were included in this review. The sample sizes ranged from 50 to 20235 patients, and follow-up time ranged from 5 to 15 years. Among all studies, five were from the United States [11, 14, 15, 17, 18], three from Sweden [10, 12, 16], and two from Italy [9, 13]. The patients included in these studies had an average BMI over 35, and included women and men. Two studies focused on RYGB [14, 16], and one study on BPD [9], and one not mentioned [10], and the remaining six studies on two or more surgical types [11–13, 15, 17, 18]. Three studies followed up for 5 years (involving 282 patients), and two studies followed up for 6 years (involving 32499 patients), and one study followed up for 10 years (involving 50 patients), and one study followed up for 11 years (involving 158 patients), and one study followed up for 14 years (involving 15951 patients), and one study followed up for 15 years (involving 603 patients), and one study followed up for 20 years (involving 607 patients). Table 1 lists the main characteristics of the 10 studies deemed eligible for the meta-analysis (Table 1).

**Table 1:** Baseline characteristics of the included studies.

Auther	Year	Study country	Study design	Surgery types	Main Outcomes	Follow-up(years)	Sample size(n)	Surgery(n)	Controls(n)	BMI (kg/m^2^)	Age (years)
Fisher, D P	2018	United States	Retrospective cohort study	RYGB, SG, AGB	CVEs, death, MI, stroke	6	20235	5301	14934	≥35	19–79
Crawford, M R	2018	United States	RCT	RYGB, SG	Weight, BMI, HbA1c (%), HOMA-IR	5	95	70	25	≥35	≥45
Schauer,P R	2017	United States	RCT	RYGB, SG	MI, stroke, HbA1c (%), Fasting plasma glucose, weight, LDL, HDL, TG, SBP, DBP,	5	134	96	38	≥35	≥45
Liakopoulos, V	2017	Sweden	Prospective cohort study	RYGB	CVEs, death, MI,BMI, HbA1c (%), LDL, HDL, SBP, DBP	6	12264	6132	6132	≥40	≥45
Chen, Y	2016	United States	retrospective, cohort study	RYGB	Mixed Macrovascular complications	11	158	78	80	≥45	≥45
Mingrone, G	2015	Italy	RCT	RYGB, BPD	MI, BMI, weight, HbA1c (%), HOMA-IR, plasma glucose, HDL, LDL, TG, TC, SBP, DBP	5	53	38	15	≥35	30–60
Sjöström, L	2014	Sweden	Prospective cohort study	AGB, NAGB, VBG, RYGB	Mixed Macrovascular complications	15	603	343	260	≥34	37–60
Johnson, B L	2013	United States	Retrospective cohort study	RYGB,AGB,VBG,BPD,SG	MI, stroke	14	15951	2580	13371	≥35	≥45
Romeo, S	2012	Sweden	RCT	Not mentioned	CVEs, MI, stroke	20	607	345	262	≥40	≥45
Iaconelli, A	2011	Italy	Prospective cohort study	BPD	MI, stroke, BMI, weight, HbA1c (%), HOMA-IR, plasma glucose, HDL, TG, TC, SBP, DBP	10	50	22	28	≥35	25–60

RYGB: Roux-en-Y gastric bypass; AGB: adjustable gastric banding; SG: sleeve gastrectomy; NAGB: non-adjustable gastric banding; VBG: vertical banded gastroplasty; BPD: biliopancreatic diversion

### Results of meta-analysis for KQ1

#### Macrovascular complications

Macrovascular complications were defined as involving coronary artery diseases (such as acute myocardial infarction, unstable angina, percutaneous coronary intervention, coronary artery or bypass grafting) or cerebrovascular events (such as ischemic stroke, hemorrhagic stroke, carotid stenting, or carotid endarterectomy) identified via *ICD-9* diagnosis codes.

**Fig 2:**
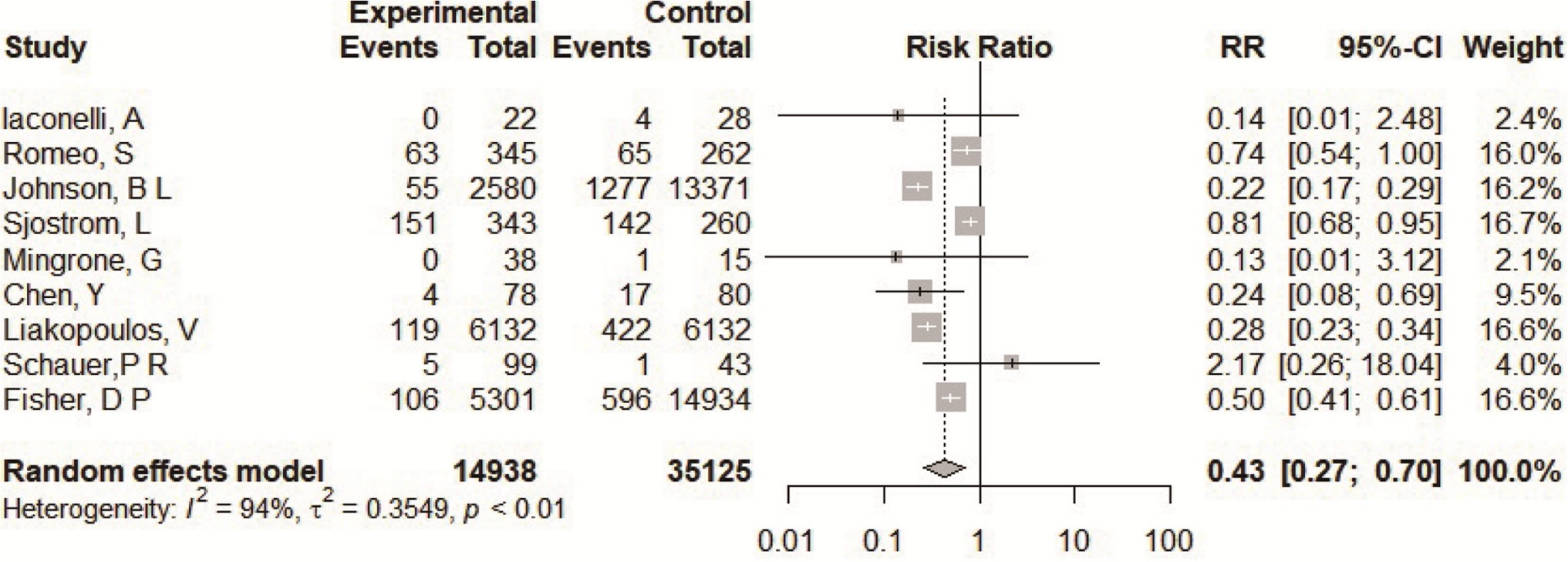
Forest plots of comparing macrovascular events between bariatric surgery and conventional medical groups.

A total of 14935 type 2 diabetes patients were from nine studies applying with accurate number of events in original articles [9–17]. Patients in the bariatric surgery group had a lower incidence of macrovascular complications compared to the conventional medical group (RR = 0.43, 95%CI = 0.27~0.70) (Fig 2). The heterogeneity among these studies was significant (*I*^*2*^ = 94%). The Baujat plot showed that there were three studies that contributed significantly to the heterogeneity [11, 12, 16], while one had a large impact on heterogeneity and influence of overall research results [12], the other two studies only contributed to heterogeneity [11, 16] (S2 Fig). The funnel plot for RRs showed no evidence of obvious asymmetry (*P* = 0.898) S2 Fig). We performed a sensitivity analysis by omitting one study at a time and calculating the pooled RRs for the remaining studies. The results show that none of the individual studies influenced the final conclusion (S2 Fig). Subgroup analyses based on the study design suggested that heterogeneity decreased for RCTs (RR = 0.75, 95% CI = 0.46~1.22, with *I*^*2*^ of 4.8%) (S3 Fig).

**Fig 3:**
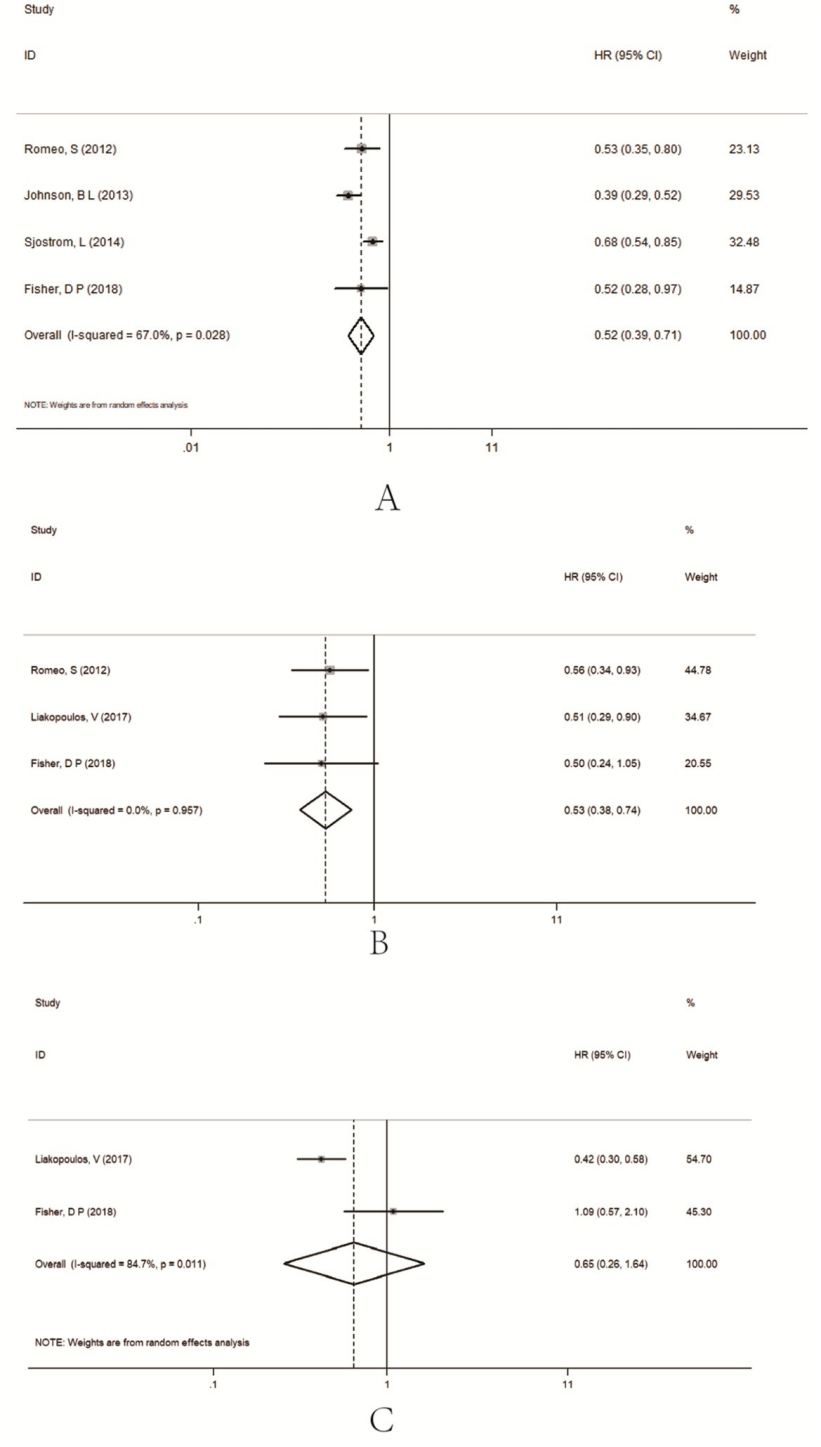
Forest plots of comparing macrovascular complications (A), cardiovascular events (B), death (C) between bariatric surgery and conventional medical groups based on adjusted HR for the original study.

A total of 8569 type 2 diabetes patients were from four studies with an adjusted HR and 95%CI in the original studies [10–12, 17]. The result with adjusted HRs showed that the incidence of macrovascular diseases was lower in the bariatric surgery groups (HR = 0.52, 95%CI = 0.39~0.71) (Fig 3). There was significant heterogeneity among the studies (*I*^*2*^ = 67%).

#### Cardiovascular events

A total of 8569 type 2 diabetes patients were from three studies with an adjusted HR and 95%CI in original reports [10, 16, 17]. The result with the adjusted HRs showed that the incidence of CVEs was lower in the bariatric surgery groups (HR = 0.52, 95%CI = 0.39~0.71) (Fig 3). There was no heterogeneity among the studies (*I*^*2*^ = 0%).

#### All-cause mortality

A total of 11433 type 2 diabetes patients were from two studies with an adjusted HR and 95%CI in original reports [16, 17]. The result with adjusted HRs showed that the incidence of all-cause mortality was no different between the two groups (HR = 0.65, 95%CI = 0.26~1.64) (Fig 3). There was significant heterogeneity among the studies (*I*^*2*^ = 84.7%).

#### Myocardial infarction (MI)

**Fig 4:**
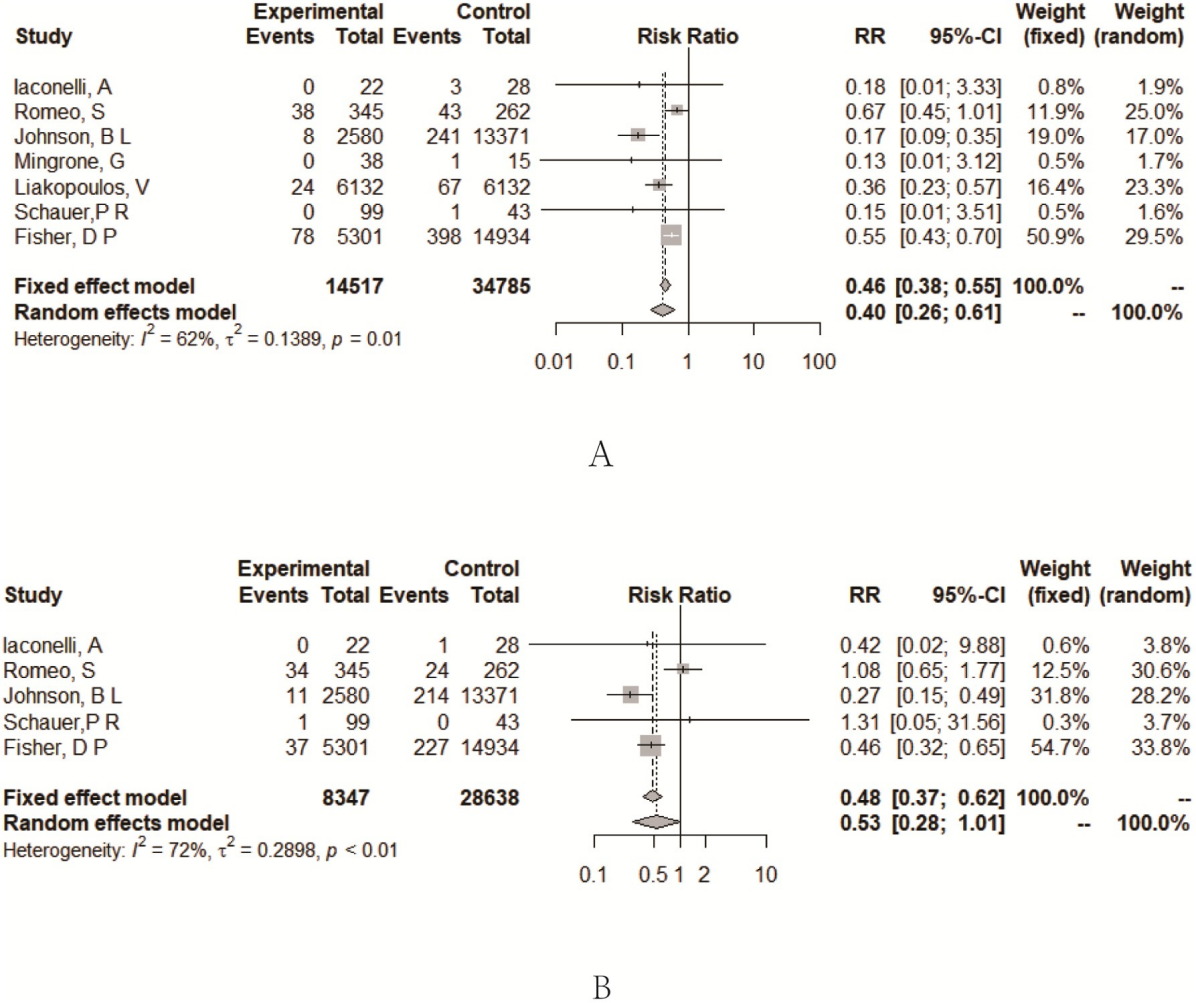
Forest plots of comparing myocardial infarction (A), stroke (B) between bariatric surgery and conventional medical groups.

A total of 14514 type 2 diabetes patients were from seven studies with an accurate number of events in the original reports [9–11, 13, 15–17]. Patients in the bariatric surgery group had a lower incidence of MI compared to the conventional medical group (RR = 0.40, 95%CI = 0.26~0.61) (Fig 4). The heterogeneity among studies was significant (*I*^*2*^ = 62%). The Baujat plot showed that there was one study that contributed significant heterogeneity [11] (S4 Fig). Subgroup analyses based on the study design suggested that heterogeneity decreased for RCTs (RR = 0.63, 95% CI = 0.43~0.93, with *I*^*2*^ of 0%) and for prospective cohort studies (RR = 0.35, 95% CI = 0.22~0.55, with *I*^*2*^ of 0%) (S4 Fig).

#### Stroke

A total of 8344 type 2 diabetes patients were from five studies with an accurate number of events in the original reports [9–17]. There was no difference between the two groups (RR = 0.53, 95%CI = 0.28~1.01) (Fig 4). The heterogeneity among the studies was significant (*I*^*2*^ = 72%). The Baujat plot showed that there was one study that contributed significantly both on heterogeneity and influence on overall result [10]. Subgroup analyses based on the study design suggested that heterogeneity decreased for RCTs (RR = 1.08, 95% CI = 0.66~1.77, with *I*^*2*^ of 0%) (S5 Fig), and the incidence of stroke was lower in the bariatric surgery groups for retrospective cohort studies (RR = 0.37, 95% CI = 0.22~0.63, with *I*^*2*^ of 58%).

### Results of meta-analysis for KQ2

#### Weight-related indicators

**Fig 5:**
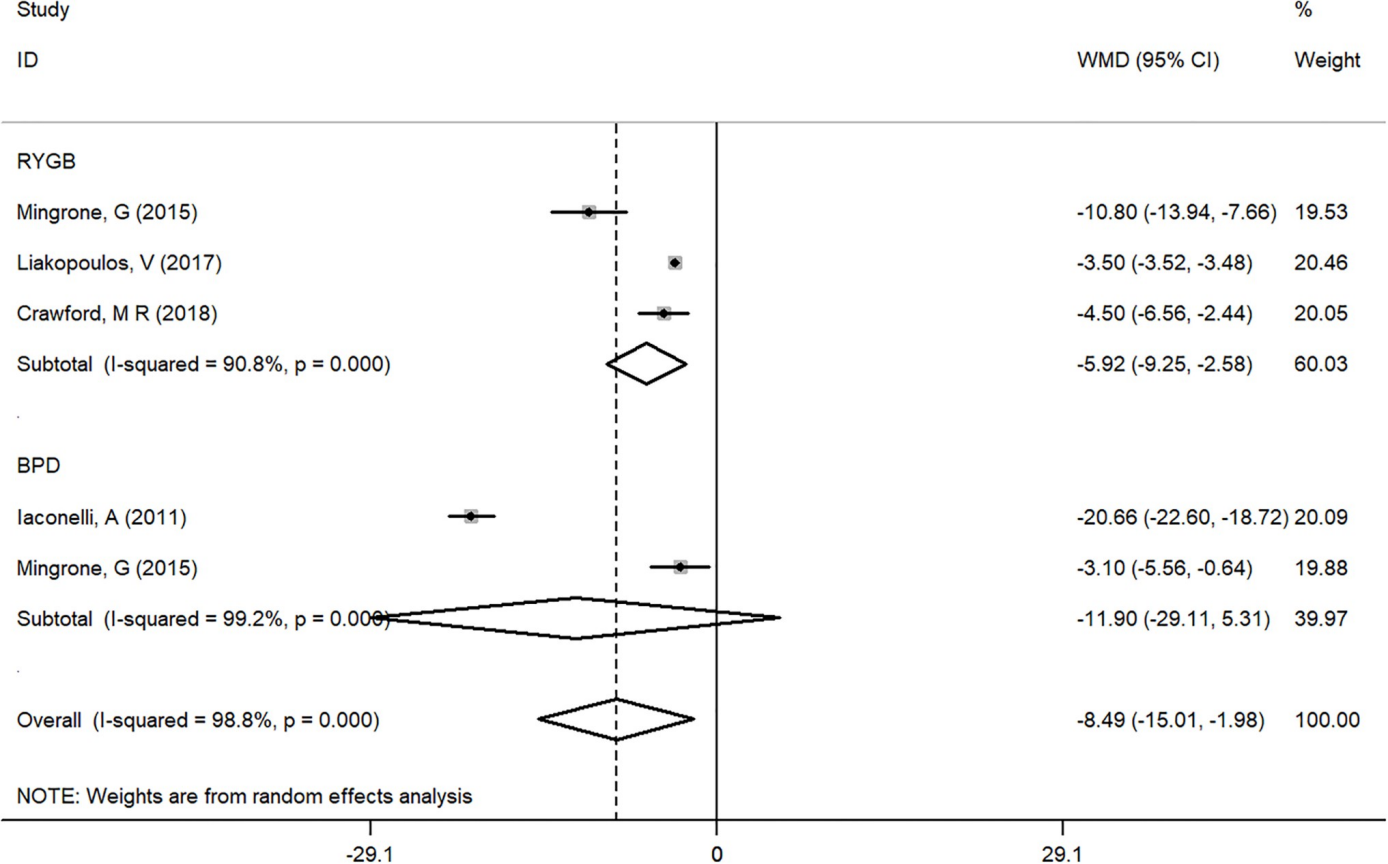
Subgroup analysis of comparing BMI between bariatric surgery and conventional medical groups by different surgery types.

A total of 6188 type 2 diabetes patients for RYGB, with 41 for BPD were from four studies [9, 13, 16, 18]. The result with WMD showed that BMI was lower in the RYGB groups (WMD = -5.92, 95%CI = -9.25~-2.58) (Fig 5) [13, 15, 18].

#### Blood glucose-related indicators

**Fig 6:**
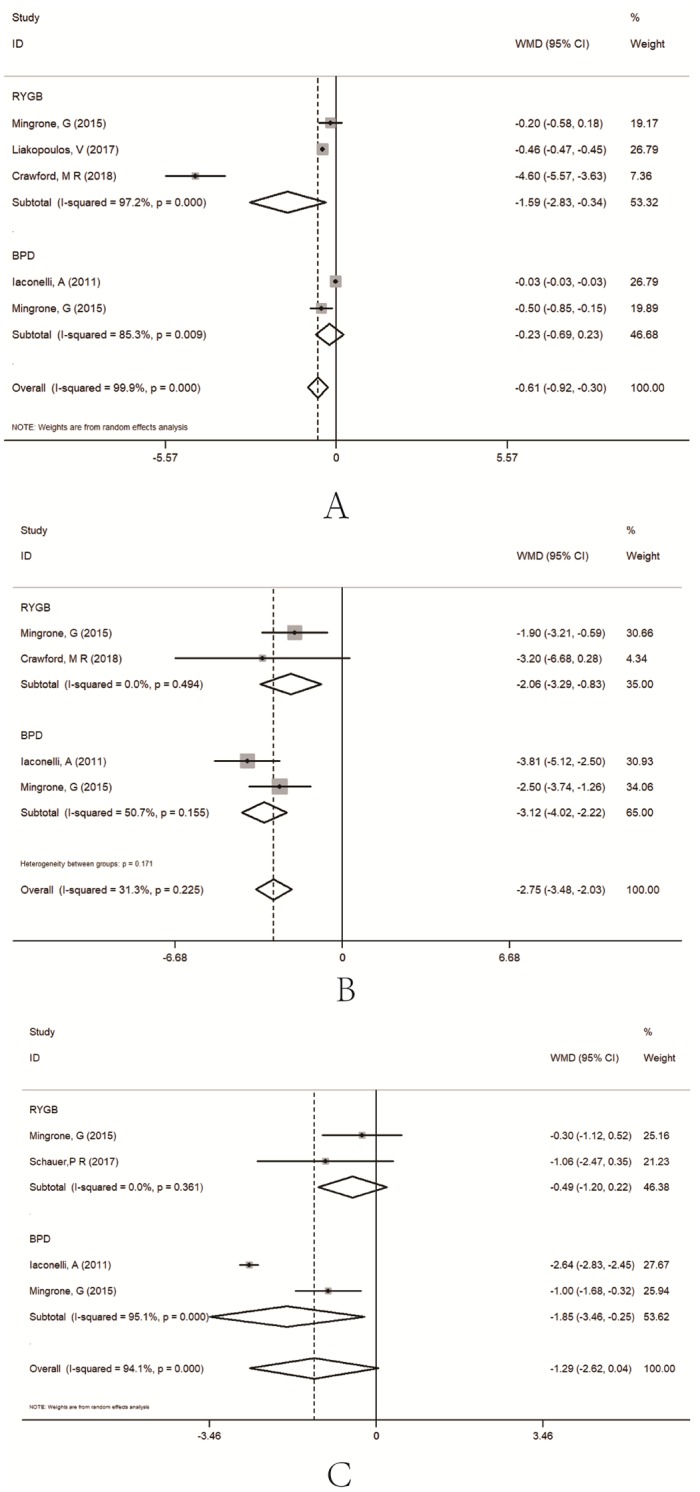
Subgroup analysis of comparing HbA1c% (A), HOMA-IR (B) and plasma glucose (mmol/L) (C) between bariatric surgery and conventional medical groups by different surgery types.

A total of 6188 type 2 diabetes patients for RYGB and 41 for BPD were from four studies [9, 13, 16, 18] (Fig 6). The result with WMD showed that HbA_1c_% was lower in the RYGB groups (WMD = -1.59, 95%CI = -2.83~-0.34) [13, 15, 18], and in the BPD group (WMD = -0.23, 95%CI = -0.69~-0.23) [9, 13].

A total of 56 type 2 diabetes patients for RYGB and 41 for BPD were from three studies [9, 13, 18]. The result with WMD showed that HOMA-IR was lower in the RYGB groups (WMD = -2.06, 95%CI = -3.29~-0.83) [13, 18], and in the BPD group (WMD = -3.12, 95%CI = -3.48~-2.03) [9, 13].

A total of 68 type 2 diabetes patients for RYGB and 41 for BPD were from three studies [9, 13, 15]. The result with WMD showed that plasma glucose was no different between the RYGB groups and the conventional medical group (WMD = -0.49, 95%CI = -1.20~0.22) [13, 15], and lower in the BPD group (WMD = -1.85, 95%CI = -3.46~-0.25) [9, 13].

#### Lipid-related indicators

A total of 6200 type 2 diabetes patients for RYGB with 41 for BPD were from four studies [9, 13, 16, 18] (S6 Fig). The result with WMD showed that HDL was higher in the RYGB groups (WMD = 0.23, 95%CI = 0.14~0.33) [13, 15, 16], and no difference in the BPD group (WMD = 0.01, 95%CI = -0.11~0.14) [9, 13].

A total of 6200 type 2 diabetes patients for RYGB were from three studies [13, 15, 16]. The result with WMD showed that LDL was no different in the RYGB groups (WMD = -0.15, 95%CI = -0.37~0.07) [13, 15, 16].

A total of 68 type 2 diabetes patients for RYGB, and 41 for BPD were from three studies [9, 13, 15]. The result with WMD showed that triglycerides was no different between the RYGB groups and the conventional medical group (WMD = -0.33, 95%CI = -0.88~0.22) [13, 15], and lower in the BPD group (WMD = -0.57, 95%CI = -0.86~-0.27) [9, 13].

A total of 103 type 2 diabetes patients for BPD were from two studies [9, 13]. The result with WMD showed that total cholesterol was lower between the BPD group than in the conventional medical group (WMD = -2.00, 95%CI = -2.56~-1.44) [9, 13].

#### Hypertension related indicators

A total of 6200 type 2 diabetes patients for RYGB and 41 for BPD were from four studies [9, 13, 15, 16] (S7 Fig). The result with WMD showed that SBP was not different between the RYGB groups (WMD = 0.00, 95%CI = -0.11~0.11) [13, 15, 16] and the BPD group (WMD = -2.66, 95%CI = -5.46~0.14) [9, 13].

A total of 6200 type 2 diabetes patients for RYGB were from three studies [13, 15, 16]. The result with WMD showed that DBP was higher in the RYGB groups (WMD = 0.90, 95%CI = 0.82~0.97) [13, 15, 16], with no difference in the BPD group (WMD = -0.34, 95%CI = -1.94~1.27) [9, 13].

## Discussion

Results of our meta-analysis show that for the severely obese T2DM patient who had their surgery five or more years prior, compared with those who undertook conventional medical therapy, the bariatric surgery group was associated with a lower risk of macrovascular complications especially in CVEs events, whereas there was no difference in all-cause mortality between the two groups. For metabolic indicators, there were better results in those who underwent surgery compared with the non-surgery group, except for blood pressure.

### For KQ1

#### Macrovascular complications

Macrovasular complications are one of the leading causes of morbidity and mortality for T2DM patients, and therefore clinical guidelines for T2DM’s management emphasize lowering macrovascular event risk factors by optimizing glycemic control, blood pressure, and serum lipid levels [26, 27]. There is ample evidence showing that intensive lifestyle intervention and pharmacotherapy are effective in helping patients to manage cardiovascular risks [28–32], however the benefits in terms of the prevention of CVD morbidity and mortality beyond those achieved through aggressive medical management of hypertension and dyslipidemia is not clear. In contrast, patients who undergo bariatric surgery had lower risk of macrovascular events and significantly better glycemic control [15, 33, 34]. A recently published meta-analysis involving five studies showed that bariatric surgery significantly reduces macrovascular complications among obese T2DM patients as compared with patients receiving only conservative medical measures (RR = 0.52, 95% CI = 0.44~0.61) [8]. In our meta-analysis of nine studies, the point estimate regarding the effects of lower macrovascular disease between two groups was smaller showing stronger protection (RR = 0.43, 95% CI = 0.27~0.70). There was significant heterogeneity across the groups involved in this meta-analysis. Based on the result of the Baujat plot, we found that the greatest heterogeneity came from the reports published in 2014 [12], 2013 [11], and 2017 [16] all belong to cohort study. Subgroup analysis based on study design showed that there was no heterogeneity in RCTs (*I*^*2*^ of 4.8%), and that there is no protective effect on macrovascular disease (RR = 0.75, 95% CI = 0.46~1.22). The prospective cohort studies were insignificant (RR = 0.42, 95% CI = 0.15~1.21), and only retrospective cohort studies showed protective effects of bariatric surgery (RR = 0.31, 95% CI = 0.16~0.62) in subgroup analyses. Because the number of RCTs and prospective cohort studies included in this meta-analysis is limited, the protective effect of bariatric surgery on macrovascular disease for severly obese patients remains to be evaluated in a high-quality prospective study. Pooling based on the number of sample cases provided by the original report couldn’t adjust the confounding factors. We merged articles provided adjusted HRs in the original text. The results showed that for macrovascular events the bariatric surgery generated the lower risk (HR = 0.52, 95% CI = 0.39~0.71), and for cardiovascular events HR = 0.53, 95% CI = 0.38~0.74. However, for all-cause mortality, bariatric surgery showed no difference with the conventional medical groups (HR = 0.65, 95% CI = 0.26~1.64). The result for death was inconsistent with a previous meta-analysis (RR = 0.21, 95% CI = 0.209~0.213) [8], and the main reason was that the extracted effect indicator was different, for our meta-analysis involving the adjusted HRs applied from original papers.

#### Myocardial infarction and stroke

This is the first meta-analysis to demonstrate the protective effect against myocardial infarction and stroke provided by bariatric surgery as compared to conventional medical treatment among severely obese T2DM patients. The bariatric surgery groups had an estimated lower risk for MI (RR = 0.40, 95% CI = 0.26~0.61) as compared to non-surgical groups. We found high heterogeneity across the reports. Based on the results of the Baujat plots, we found the most important heterogeneity came from the reports published in 2013 [11]. The subgroup analyses results showed that for RCTs and prospective cohort studies heterogeneity was not significant (*I*^*2*^ of 0%), and the retrospective cohort studies were the main source of heterogeneity. For stroke, there was no difference between the bariatric groups and the non-surgical groups (RR = 0.53, 95% CI = 0.28~1.01). Subgroup analyses showed that pooling RR for retrospective cohort studies was significant (RR = 0.37, 95% CI = 0.22~0.63). However, because of the very limited number of studies included, the conclusion needed to be further confirmed.

### For KQ2

The current evidence suggests that bariatric surgery is helpful for obesity patients (BMI of 30–35) with T2DM and is associated with greater short-term weight loss and better intermediate glucose outcomes [3, 35]. Although the guide recommended bariatric surgery is beneficial for patients above 35 with T2D, there was insufficient evidence to address these population, especially for a long-term effect beyond 5 years followed up. The systematic review published in 2013 confirmed that bariatric surgery is associated with lower weight and better glycemic control for the short term (follow-up during 1 to 2 years) than that of non-surgical groups. The objective of our study was to evaluate the long-term outcomes of diabetes comparing bariatric surgery to non-surgical treatments in severely obese patients (BMI ≥ 35) with T2DM. We evaluated the overall effect and different surgical approaches including RYGB, BPD, and SG. The results show that the bariatric surgery group had lower BMI (WMD = -8.49, 95% CI = -15.01~-1.98), especially in the RYGB group (WMD = -5.92, 95% CI = -9.25~-2.58), whereas the BPD group was not significantly different from standard medical treatment.

For HbA1c (%), only the RYGB groups showed a lower level as comparted to the control group; for HOMA-IR, the BPD groups showed a smaller effect than the RYGB groups; and for plasma glucose, the BPD groups had a significant effect. Therefore, the different surgical methods gave different results for the three glycemic control indicators. The results showed that RYGB had a better performance in HDL, whereas for triglycerides and total cholesterol, BPD could effectively reduce the indicators.

### Strengths and limitations

Given the lack of evidence on the long-term benefit of bariatric surgery for severely obese patients with type 2 diabetes, there is an urgent need for an assessment of this issue. This meta-analysis evaluated the long-term effects of different types of bariatric surgery on prognostic indicators and for MI and stroke incidence in T2DM. It was found that the different surgical methods have different effects on metabolic indicators. We performed a subgroup analysis and found that the retrospective cohort study contributed high heterogeneity. We merged the adjusted HR values provided in the original manuscript to eliminate the effects of some confounding factors, and the results were more realistic and reliable. However, there are several limitations that should be considered. First, the heterogeneity is a potential problem when interpreting the results of meta-analyses [36]. In our meta-analysis, high heterogeneity persisted in studies. The first reason of high heterogeneity was the involving studies in this meta-analysis focused on different clinical outcomes, which means that there were differences in patients included in each of the studies. And then, the diagnostic criteria for macrovascular complications and the surgical approach varied considerably among involved studies, which potentially contributed to the high heterogeneity. Second, because of the lack of a method to identify whether preoperative and postoperative adjuvant treatments were different, it is not possible to confirm that changes in metabolic indicators were indeed caused by surgery. Third, due to the low number of RCTs and cohort studies, we couldn’t carry out the analysis of publication bias for every question. Third, only some studies provide corrected HR values, so there was limited effective adjustment for confounding factors.

## Conclusion

In conclusion, bariatric surgery could significantly reduce long-term macrovascular complications along with greater weight loss and better intermediate glucose outcomes among severely obeseT2DM patients when compared to patients receiving only conservative medical measures.

## Supporting information

S1 Fig Cochrane risk of bias of randomized clinical trials of bariatric surgery. (TIF)

S2 Fig Funnel plot (A), Baujat plot (B), and Sensitivity analysis plot (C) of macrovascular complications between bariatric surgery and conventional medical groups. (TIF)

S3 Fig Subgroup analysis of comparing macrovascular events between bariatric surgery and conventional medical groups. (TIFF)

S4 Fig Subgroup analysis of comparing MI (A) between bariatric surgery and conventional medical groups by different surgery types. Baujat plot for MI (B).
(TIF)

S5 Fig Subgroup analysis of comparing stroke (A) between bariatric surgery and conventional medical groups by different surgery types. Baujat plot for MI (B).
(TIF)

S6 Fig Subgroup analysis of comparing HDL (mmol/L) (A), LDL (mmol/L) (B) and Triglycerides (mmol/L) (C) and total Cholesterol (mmol/L) (D) between bariatric surgery and conventional medical groups by different surgery types. (TIF)

S7 Fig Subgroup analysis of comparing SBP (mmHg) (A), DBP (mmHg) (B) between bariatric surgery and conventional medical groups by different surgery types. (TIF)

S1 Table OTTAWA quality assessment scale cohort studies. (DOCX)

S1 File PRISMA Checklist. (DOC)
