# Supplementary figures and images for: Long-term outcomes of macrovascular diseases and metabolic indicators of bariatric surgery for severe obesity type 2 diabetes patients with a meta-analysis
Source: PLoS One. 2019 Dec 3;14(12):e0224828. doi: 10.1371/journal.pone.0224828 (PMC6890174; doi:10.1371/journal.pone.0224828)

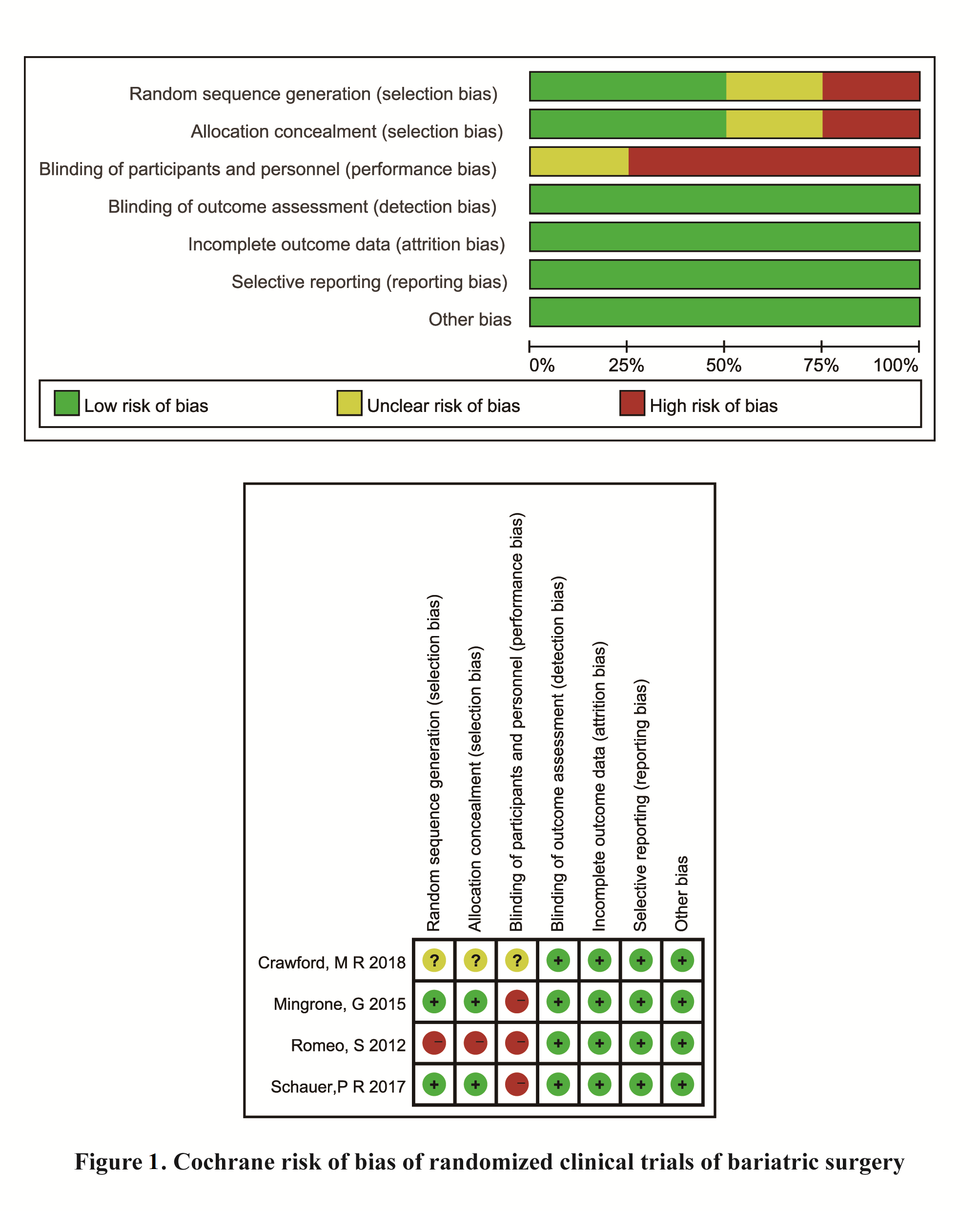

Supplement: S1 Fig — (TIF) [file pone.0224828.s001.tif]

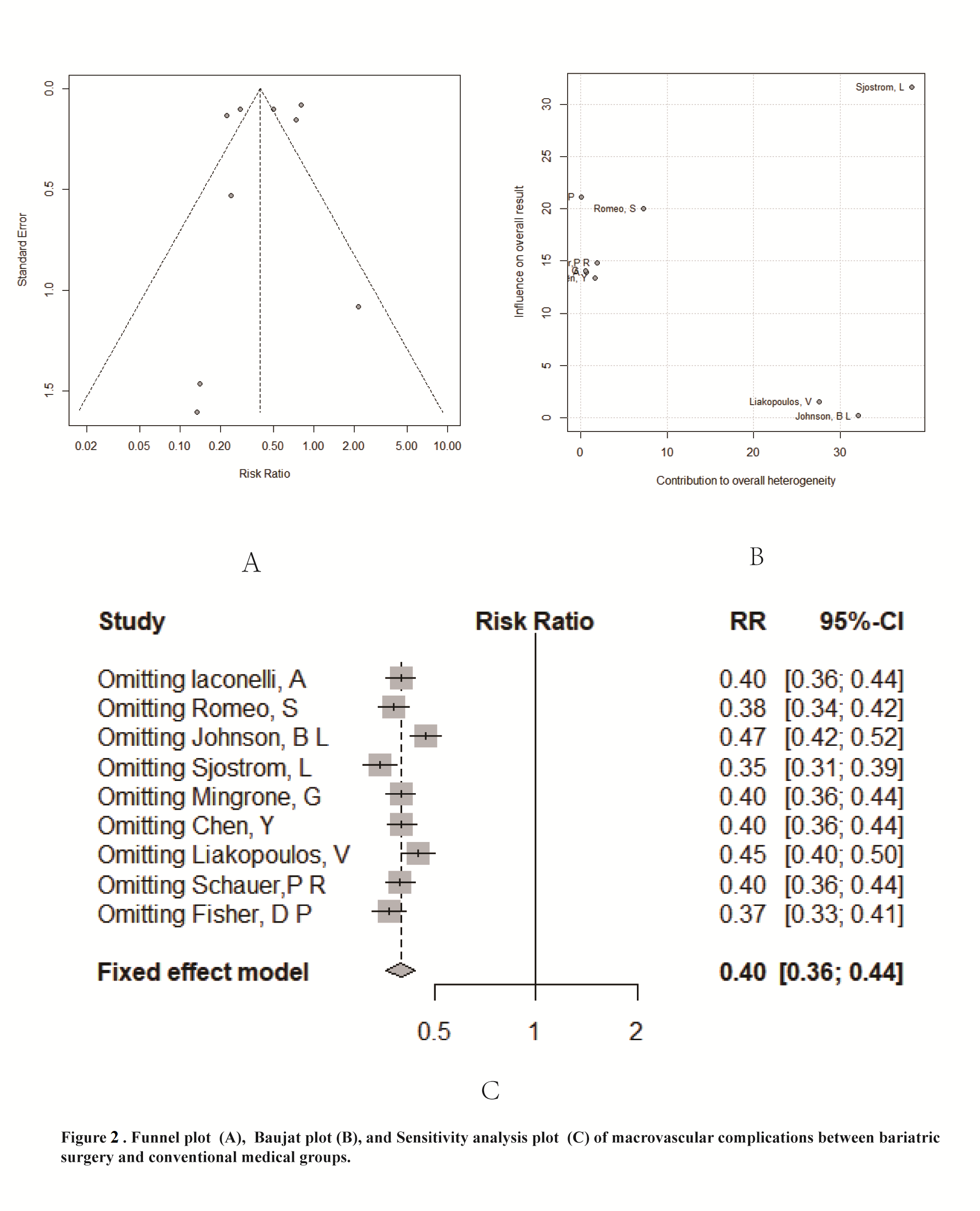

Supplement: S2 Fig — (TIF) [file pone.0224828.s002.tif]

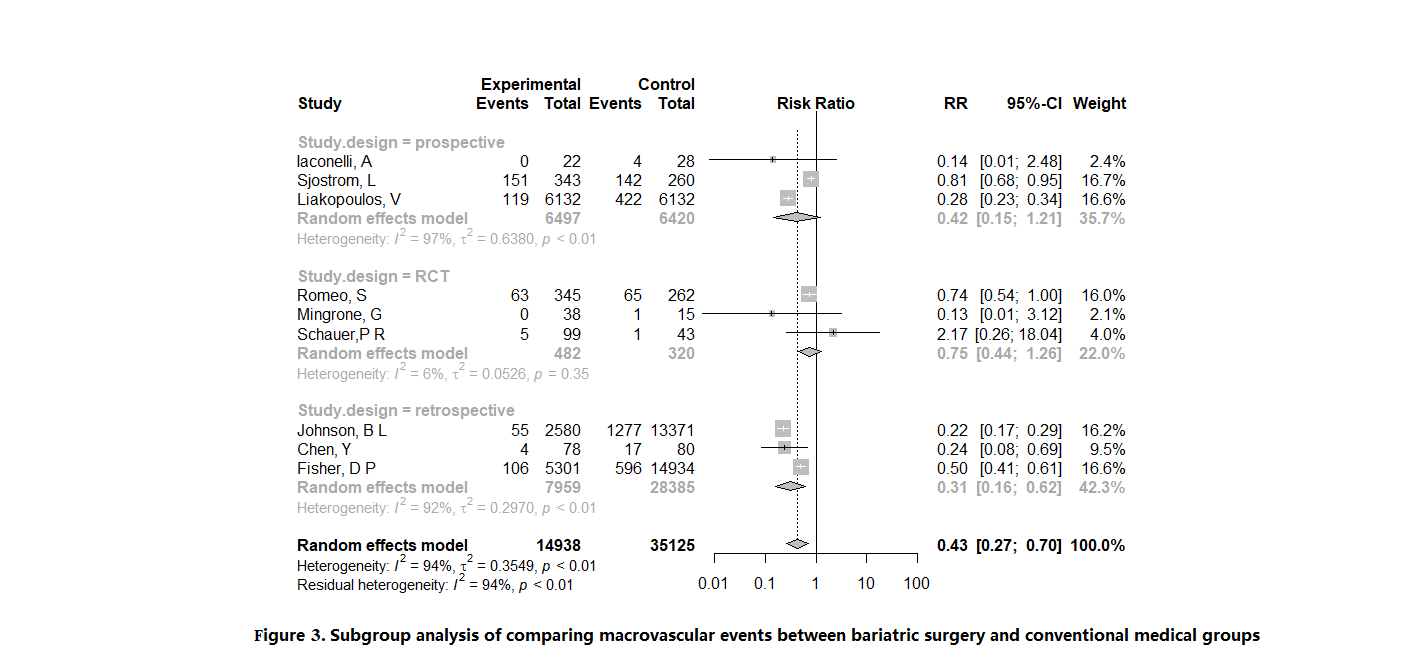

Supplement: S3 Fig — (TIFF) [file pone.0224828.s003.tiff]

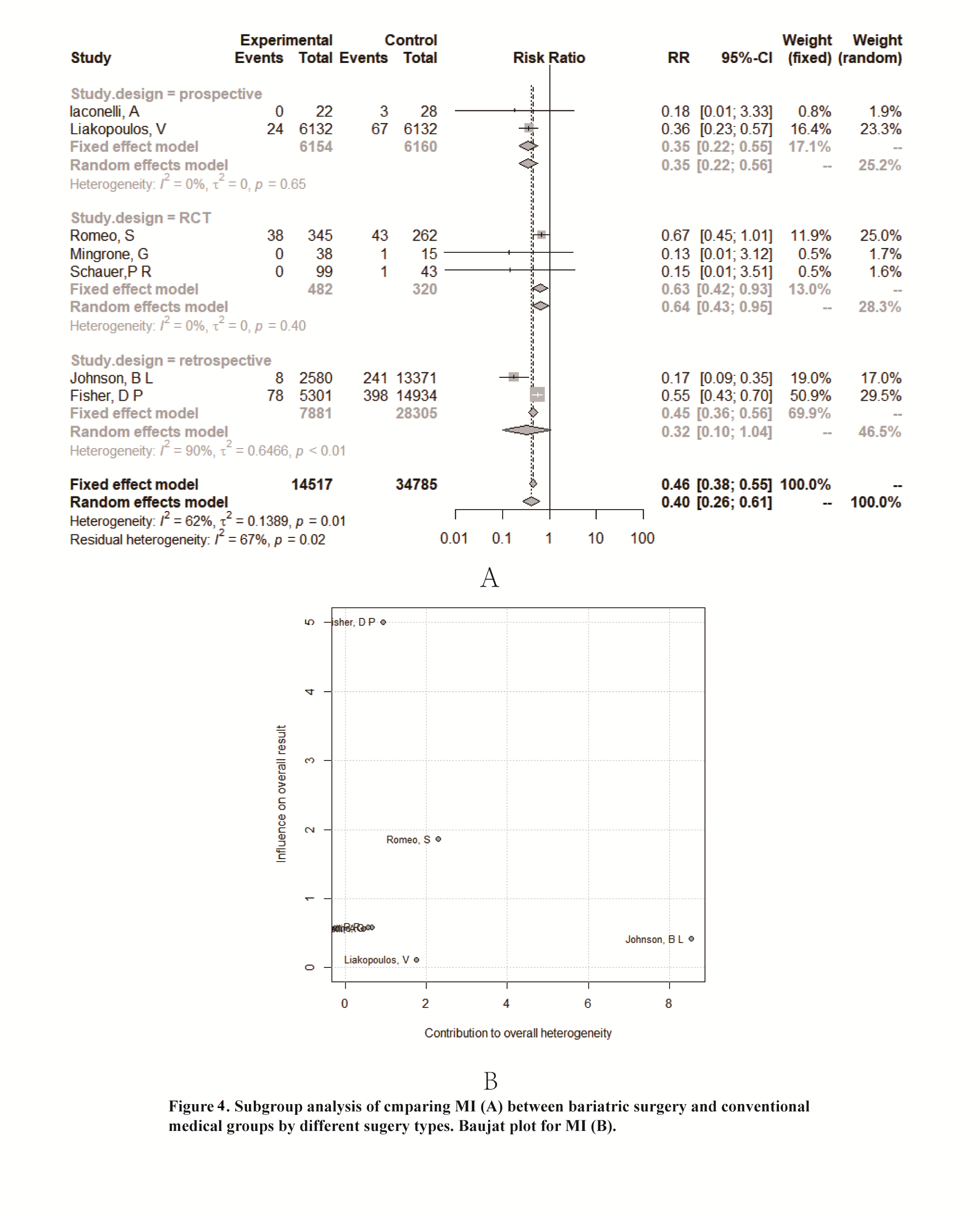

Supplement: S4 Fig — Baujat plot for MI (B). (TIF) [file pone.0224828.s004.tif]

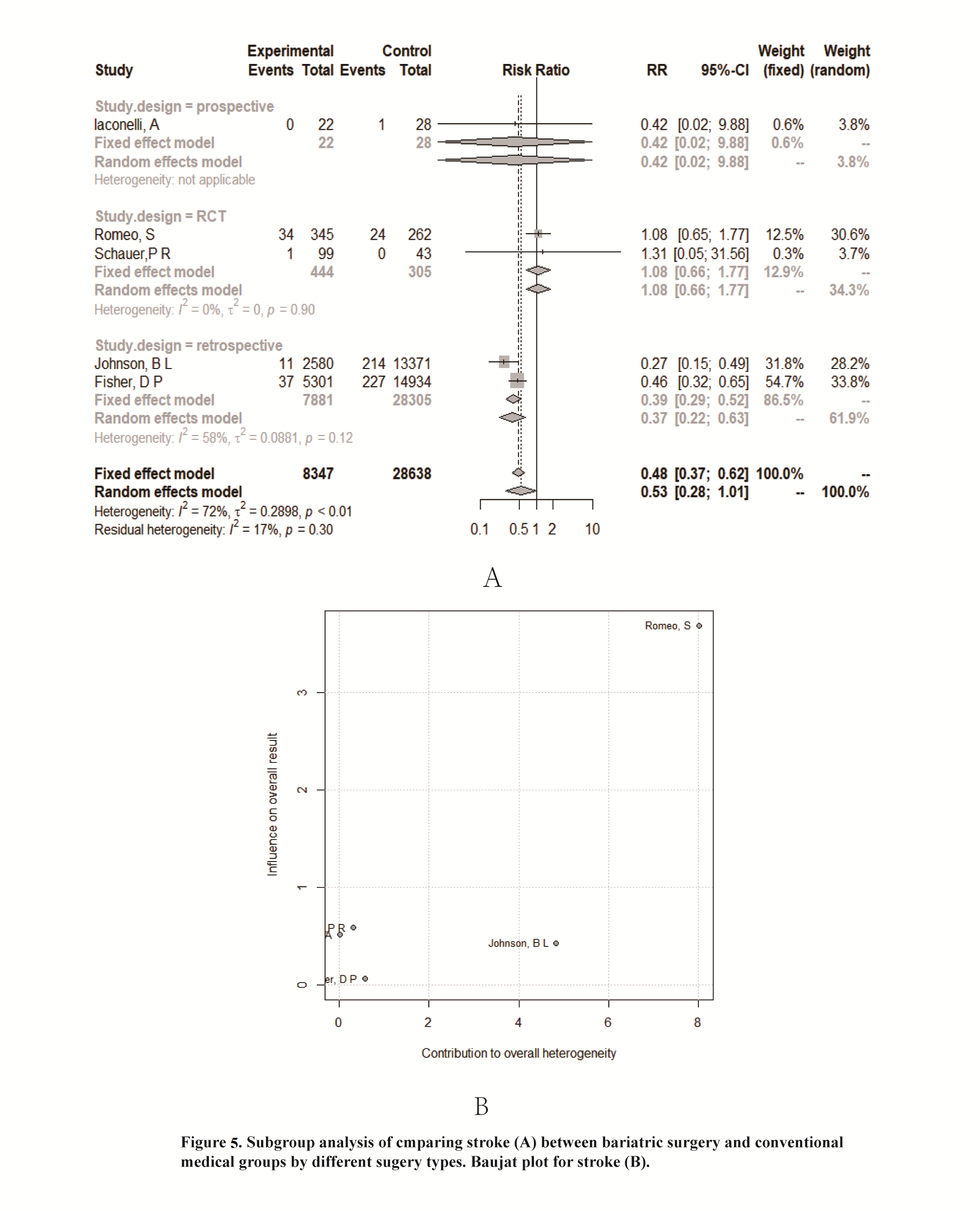

Supplement: S5 Fig — Baujat plot for MI (B). (TIF) [file pone.0224828.s005.tif]

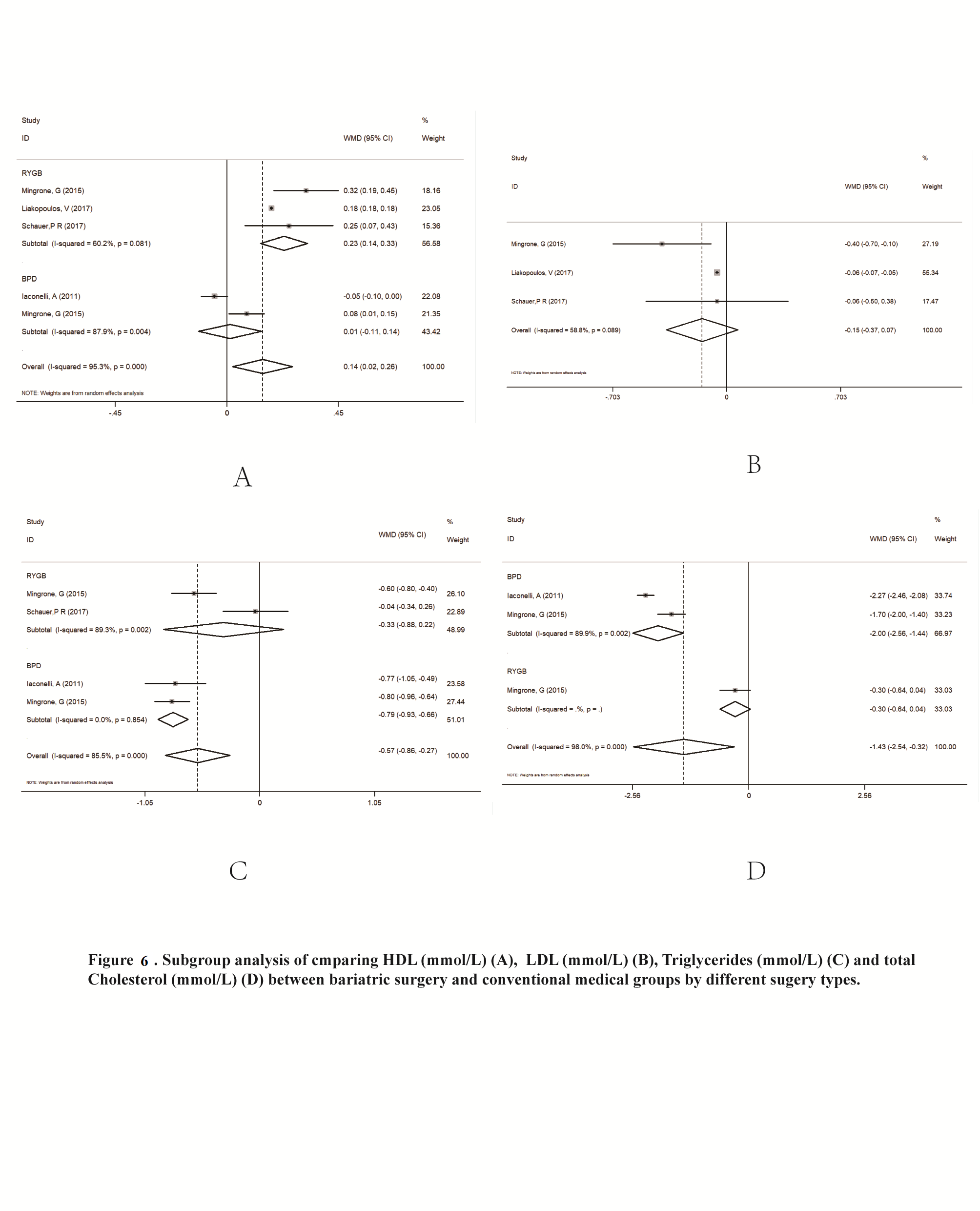

Supplement: S6 Fig — (TIF) [file pone.0224828.s006.tif]

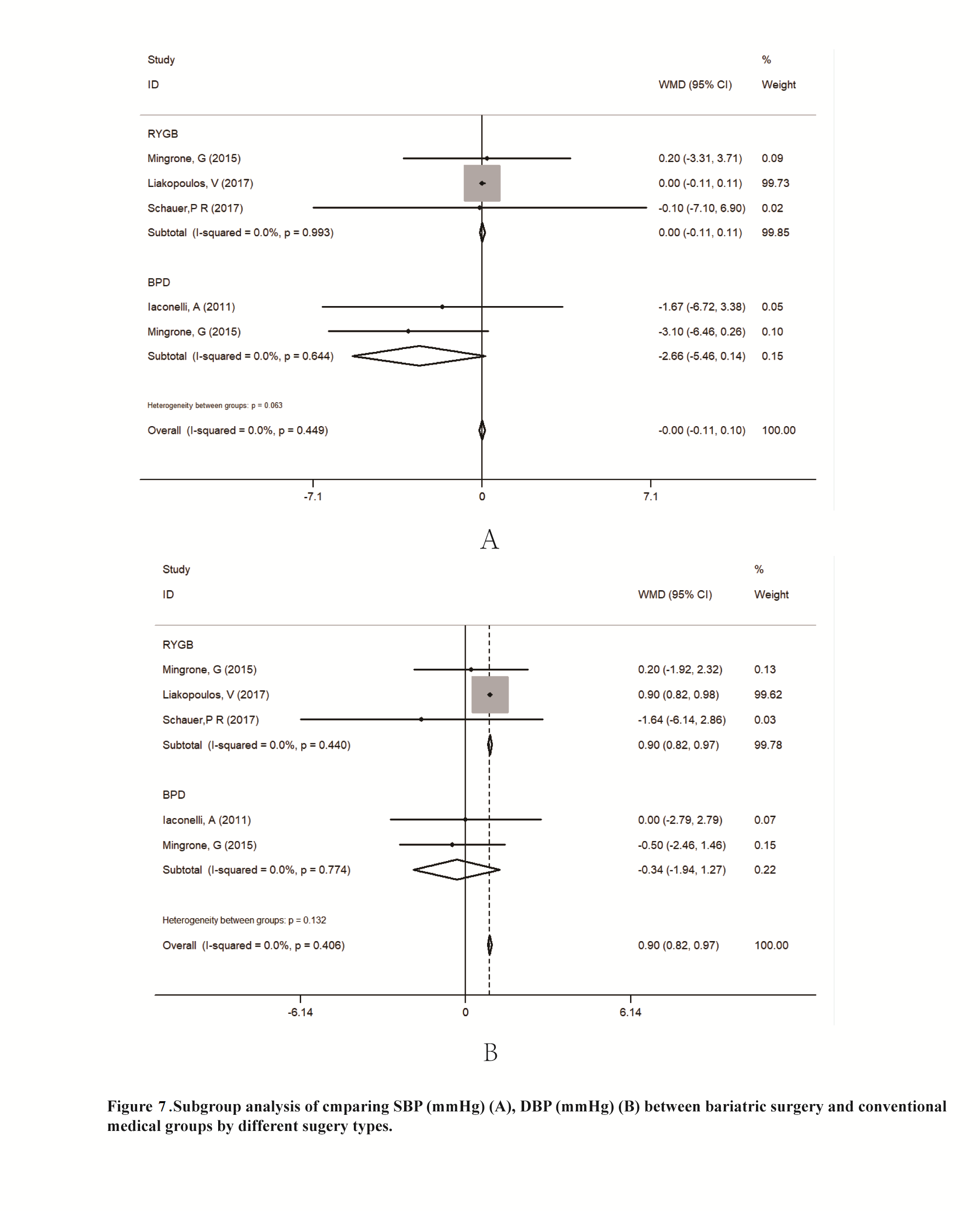

Supplement: S7 Fig — (TIF) [file pone.0224828.s007.tif]
