# Supplementary material for: Long-term outcomes of macrovascular diseases and metabolic indicators of bariatric surgery for severe obesity type 2 diabetes patients with a meta-analysis
Source: PLoS One. 2019 Dec 3;14(12):e0224828. doi: 10.1371/journal.pone.0224828 (PMC6890174; doi:10.1371/journal.pone.0224828)
Supplement: S1 Table — (DOCX) [file pone.0224828.s008.docx]

| **S1 Table. OTTAWA QUALITY ASSESSMENT SCALE COHORT STUDIES** | | | | | | |  |
| --- | --- | --- | --- | --- | --- | --- | --- |
| **Author** | **Year** | **Study country** | **Study design** | **Selection** | **Comparability** | **Outcome** | |
| Fisher, D P | 2018 | United States | Retrospective cohort study | 4 | 2 | 2 | |
| Liakopoulos, V | 2017 | Sweden | Prospective cohort study | 3 | 2 | 2 | |
| Chen, Y | 2016 | United States | retrospective, cohort study | 2 | 2 | 2 | |
| Sjöström, L | 2014 | Sweden | Prospective cohort study | 2 | 2 | 2 | |
| Johnson, B L | 2013 | United States | Retrospective cohort study | 2 | 2 | 2 | |
| Iaconelli, A | 2011 | Italy | Prospective cohort study | 2 | 2 | 3 | |
